# Supplementary material for: Are carbon emissions trading and green financial instruments synergistic? -Comprehensive quantitative research based on content analysis
Source: PLoS One. 2024 Mar 7;19(3):e0298601. doi: 10.1371/journal.pone.0298601 (PMC10919601; doi:10.1371/journal.pone.0298601)
Supplement: S1 Data — (ZIP) [file pone.0298601.s001.zip › Data/地级市Z_F工作报告.zip!/╣π╢1⁄2/╘╞╕í╩╨/└┤╫╘╣1⁄2╓┌║┼í╛┬φ┐╦╩2╛▌═°í┐_2014.docx]

各位代表：

我代表市人民政府向大会作政府工作报告，请予审议，并请政协各位委员和其他列席人员提出意见。

2013年工作回顾

2013年，是我市抢抓机遇、争先进位，在复杂形势下营造新优势、实现新突破的一年。一年来，市政府在省委、省政府和市委的正确领导下，在市人大及其常委会和市政协的监督支持下，紧紧围绕“跨越赶超、科学崛起”和建设美丽幸福新云浮目标，抓住省委、省政府促进粤东西北地区振兴发展决策部署实施的机遇，守住社会稳定和生态保护两条底线，突出交通基础设施建设、产业园区建设和中心城区扩容提质三个抓手，较好完成了市五届人大三次会议确定的各项目标任务。

——经济发展持续提速。

预计全市实现地区生产总值602.3亿元，增长13.3%；人均生产总值增长12.2%；规模以上工业增加值192.06亿元，增长26%；地方公共财政预算收入45.75亿元，增长24.45%；固定资产投资总额623.38亿元，增长34.4%；社会消费品零售总额204.02亿元，增长13.1% ;外贸进出口总额15.81亿美元，增长8.5%；实际利用外资1.11亿美元，增长4.9%；12月末，金融机构本外币存款余额744.9亿元、贷款余额471.52亿元，分别增长13.1%和19.2%；城乡居民人均收入增长11.5%。

——转型升级步伐加快。

积极推进重点工程项目建设，千方百计发展新兴产业，力促转型升级。大西关经贸建设活动和第十届石展会暨第四届石文化节圆满成功。全市新引进合同项目252个，计划总投资685亿元,投资超10亿元的项目有17个。107项重点工程项目完成投资239.68亿元，完成年度计划的105%，其中列入省的28项重点建设项目完成投资124.57亿元，完成年度计划的120.6%。四大产业园区实现规模以上工业总产值255.1亿元，占全市的33.2%；实现规模以上工业增加值63.62亿元，占全市的33.1%；新增基础设施投资27.17亿元，投资额为历年最大。累计引入项目381个，总投资683.43亿元，目前建成投产项目231个，完成投资213.98亿元；在建项目73个，投入资金62.63亿元。佛山（云浮）产业转移工业园年内完成投资37亿元，引进产业项目8个，高丘六和、爱德克斯、远大药业和广云铝板带等项目建成投产；佛山顺德（新兴新成）产业转移工业园年内完成投资12亿元、引进项目6个，摩德娜机械、恒事泰金属制品、大华农饲料添加剂等项目建成投产；云浮双东环保工业园被批准为“广东省循环经济工业园”，年内完成投资14.8亿元、引进项目27个，天创双东环保开发有限公司部分项目、友源电气、骏鑫化工等项目建成投产，中顺纸业（罗定）基地首期两条生产线即将投产；云浮循环经济工业园年内完成投资7.27亿元，新签约项目20个，计划总投资53.54亿元，青洲水泥（云浮）有限公司首条日产4500吨熟料水泥生产线建成投产。汽车配件、生物制药、新材料等新兴产业初具规模。全国“三网融合”试点城市和广东省“智慧城市”建设深入推进，成功获得省级IPTV平台测试资格，信息产业集聚发展初显成效。实施“还珠工程”，优化提升市场主体比例结构，引导2314户个体工商户升级登记为企业，网报企业新增221家。市场主体突破6万大关，达到6.6万户，公司、企业登记数突破1万大关，增速为历年来最快。新增规模以上工业企业89家，实现规模以上工业总产值51.4亿元，占规模以上工业总产值的6.7%，拉动规模以上工业增长8.83个百分点；先进制造业实现总产值148.15亿元，增长28.5%，占规模以上工业总产值的19.2%；高技术产业实现总产值51.43亿元，增长20.5%，占规模以上工业总产值的6.7%。万元工业增加值能耗下降20.24%。

——交通建设成效显著。

全市交通基础设施建设完成65.21亿元，完成年度计划的107.3%。全年安排13项重点交通建设项目完成60.1亿元，完成年计划的101.4%，年交通项目数量、投资额均创历史新高。其中高速公路完成32亿元，一般公路完成23.07亿元，铁路、水路和站场完成10.14亿元。列入省计划的重点项目全部完成年度建设任务，云罗高速建成通车，南广铁路云浮段完成全线铺轨，江罗高速完成14.5亿元，阳罗高速完成5.5亿元。农村公路建设完成418公里任务。公路密度从上年的每百平方公里97.8公里提高到101.2公里。

——扩容提质扎实推进。

《云浮新区发展总体规划（2013-2030）》获省政府批复同意实施，新区规划面积535.5平方公里，范围包括安塘、都杨、腰古、思劳4个镇（街）。云浮西江新城中央商务区控制性详细规划及城市设计已经完成；其他重要片区的控制性详细规划编制工作陆续展开；新城水景中轴线及滨江公园城市（景观）设计方案国际竞赛已经完成，现正进行深化设计。目前，新区已引进项目131个，计划总投资730.56亿元，落实融资项目7个，金融机构融资额43.6亿元。去年8月以来，新增动工项目38个，计划投资总额266.02亿元；新增竣工项目11个，投资总额37.17亿元。交通基础设施、城市生态景观、民生服务、公用设施、高铁中央商务区、特色小镇等20个重点项目年度完成投资36.36亿元。新区碧桂园城市花园、宝能城市广场综合体、远大美域特色小镇和五星级酒店加快建设，华立城市学院投入使用并正式招生。新区土地治理及大涌河流域水利工程、生态景观林建设等3个生态环保项目有序开展。大力推进中心市区“三旧”改造，改造项目29个面积1100.53亩。累计投入20多亿元改造和建设市政设施，提升城市功能，创建国家卫生城市工作顺利通过国家技术总评审，省级卫生镇创建工作取得新的突破；金丰路、浩林西路等多条道路实施改造和贯通；创建省文明城市取得良好成效。同时，各县（市、区）扩容提质工作扎实推进。罗定市城市总体概念规划修编全面完成，中心城区规划建设面积扩大一倍；新兴县动工建设惠能纪念广场、新体育中心、新人民医院、五星级酒店等项目，并加快推进市政道路改造，城市面貌大大改善；郁南县全面贯通九星大道延长线，拉开新区发展架构；云安县抓好青洲大道、500千伏输变电工程等基础设施建设，着力提升城市承载力。

——综合改革亮点纷呈。

农村改革深入推进。加快建设国家农村改革试验区，探索创新现代农业经营制度和“五位一体”农村改革取得实质性成效，已接受国家农业部的中期评估。多种形式创新现代农业经营体制，温氏集团“公司+现代家庭农场”模式进一步推广，在全国建立了2047个现代家庭农场，物联网智能养殖项目进入实际应用；全市有9家公司组织实施“公司+合作社（理事会）+农户”、13家合作联社组织实施“合作社+合作社”经营模式。农业产业化发展加快，新发展各类农业产业化组织1300个，其中市级龙头企业9个、农民专业合作社1134个，新兴县被农业部认定为全国农业产业化示范基地。积极推进社会综合服务网络、农村“三资”监管和土地流转平台建设，建立县、镇、村三级社会综合服务平台192个，镇级资产资源交易平台86个，县、镇两级农村土地流转平台34个，新兴县和云安县所有镇均建立了农村土地流转中心。创建国家农产品质量安全监管示范市，在全省率先实现监管公共服务机构100%覆盖到镇。全力推进农业标准化示范区建设，建设了广东新兴肉鸡标准化示范区、郁南县无核黄皮标准化示范区和国家级沙糖桔标准化示范县等5个标准化示范基地。

信用云浮有序开展。扎实推进省社会信用体系建设试点工作，加快建设全省农村金融改革创新综合试验区。出台了《云浮市农户小额贷款成本分担和风险补偿基金管理暂行办法》，全面建立和完善农户信用贷款风险补偿机制。围绕信用云浮建设，全面深入开展“两建”工作，在全省考核评比中获得良好成绩。市县两级征信中心企业信用信息系统已对全市9.14万家企业和个体工商户建立信用档案，农村信用信息系统已对全市57.67万户农户建立家庭信用档案。建成信用村812条，占行政村总数的95.86%。继续探索农民建房贷款试点工作，有324户农户获得农村建房贷款授信1416.18万元，其中195户获得建房贷款793.2万元。建设石材中小微企业信用合作平台和进出口企业信用合作平台，促进实体经济发展。提高跨境人民币结算比例，全年完成结算总量35.91亿元，占同期跨境收支额的33.34%。

行政体制改革继续深化。进一步统筹食品药品监管、医疗卫生和计划生育服务资源，整合食安办和食品药品监管、工商、质监、卫生等部门的食品安全监管、药品管理职能以及经信部门的酒类流通监管职能。不断深化行政审批制度改革。大力清理行政审批事项，全市共调整压减行政审批事项1491项，调整压减率达43.96%。出台了《行政审批事项目录管理办法》，启动商事登记制度改革，降低准入门槛、提高登记效率、强化后续监管。积极推行政府重大行政决策专家咨询论证，对涉及全市经济社会发展全局、与人民群众利益密切相关的重要工作事项，组织开展咨询论证12场次，参与专家122人次。建立完善电子监察系统平台，进一步强化行政效能电子监察。网上办事大厅和基层便民服务站建设走在全省前列，比省的要求提前半年开通至县一级，提前三年开通镇一级，同时建成村级基层便民服务系统，实现市、县、镇、村四级联网办事，全省网上办事大厅建设现场会在我市新兴召开。

——生态建设初见成效。

全力建设全国生态文明发展示范区。以生态文明村建设为切入点，市直副处以上、县级正科以上干部挂钩联系一条自然村的生态建设，全市共落实挂钩责任干部2945人，投入资金4.24亿元，其中外出乡贤投入9900万元，建设生态文明村2968条，覆盖率达33.8%。农村生活垃圾治理工作扎实推进，“一县一场、一镇一站、一村一点”建设任务基本完成，全省农村生活垃圾处理工作现场会在罗定召开。建设省立绿道169.2公里。气象现代化建设加快推进，市区新空气质量监测系统投入试运行，已具备发布PM2.5等数据能力，环境质量持续改善。全市空气质量保持在国家二级标准以上，市区空气质量优良率、饮用水源水质达标率均为100%，西江、南山河、南江河、新兴江等主要河流水质保持优良。狠抓治污减排，列入全省减排计划的52个项目全面完成。城镇生活污水集中处理率达72.5%，城市生活垃圾无害化处理率达80%。治洪治涝保安工程、农田水利建设工程、村村通自来水工程、农村安全饮水等民生水利工程建设顺利推进，累计完成投资4.1亿元。“三防”和救灾复产工作扎实推进。完成森林碳汇工程建设13.81万亩，建设林业生态文明“万村绿”村庄114条，完成任务的114%，森林覆盖率达68.4%。

——社会民生持续改善。

民生保障水平提高。全市民生类支出完成77.27亿元，同比增长18.01%，占公共财政预算支出的72%。十件民生实事全面完成，解决了一批教育、就业、医疗、住房等领域的难点热点问题。城镇新增就业3.99万人，完成年度计划的128%，城镇登记失业率2.37%。超额完成社会保险扩面征缴目标任务，社保卡基本覆盖常住人口。城乡居民基本医疗保险住院医疗费政策范围内报销比例达到75%，位居全省同类地区前列。城乡居民社会养老保险基础养老金由每人每月55元提高至65元，惠及全市32.09万参保人。职工基本医疗保险住院医疗费政策范围内报销比例达到85%以上。人力资源社会保障基层服务平台覆盖全市63个镇（街）。民政优抚工作稳步推进，全市抚恤补助均达到或高于省定标准。扶贫开发“双到”工作共投入帮扶资金2.19亿元，其中佛山市对口帮扶资金1.12亿元。完成2815户农村低收入住房困难户住房改造建设任务。动工建设保障房项目10个，基本建成1215套。新建平价商店56家，完成率达124%。实施临时价格补贴机制，向30万多人次低收入和特殊困难群众发放价格补贴878.82万元。

医疗卫生、文化科教全面发展。医疗卫生建设和服务水平显著提高。全面完成15项医改重大项目和基本公共卫生服务项目任务，其中，63间基层医疗卫生机构卫生监督协管服务覆盖率达100%；城乡居民规范化电子健康档案建档率达97.16%、新生儿疾病筛查率达93.48%、农村改厕1.7万户;重性精神病检出率达7.79‰，我市被评为“广东省重性精神疾病管理治疗工作优秀市”;公立医院改革等七项医药卫生改革工作走在全省前列。平价医院、平价诊室、平价药包等平价医疗服务全面开展；完成对市中医院和市妇幼保健院整体搬迁，市妇幼保健院综合住院大楼动工建设，市人民医院新院二期工程顺利推进；有效防控人感染H7N9禽流感疫情，全年无发生重大疫情流行。完成年度人口与计划生育目标管理工作任务，继续稳定低生育水平，人口素质不断提高。文化建设扎实推进。建成了471个镇（村）文化站（室）和电子阅览室，完成了全市广播电视“户户通”建设任务。罗定龙龛岩摩崖石刻、郁南大湾古建筑群经国务院核定并公布为全国重点文物保护单位；新兴县图书馆被评为国家一级馆，云浮市博物馆被评为国家二级馆。云城区山城石雕工艺被列入第一批广东省非物质文化遗产生产性示范保护基地。成功举办了纪念六祖惠能圆寂1300周年暨2013广东禅宗六祖文化节。加快实施体育惠民工程，新建成11个乡镇农民体育健身广场，全民健身活动蓬勃开展。教育工作取得新成绩。23个教育创强镇（街）通过了省督前检查。全市高考本科重点上线人数627人，增幅达30%。与华南师范大学、深圳宝能集团签订教育合作项目意向。编写出版并在全市中小学使用《立德树人》一书，促进优秀传统文化进校园。建成中央福利彩票资金资助乡村学校少年宫8所。罗定职业技术学院实施“校政企”办学模式，办学水平不断提高。邓发纪念中学办学水平提升初见成效。市技工学校新校区投入使用，云浮电大建成“云浮终身学习网”。科技创新有新进步。我市被科技部评为全国科技进步先进市。高新区科技企业孵化器加快发展，已有云浮南风数字科技有限公司等20家企业入驻。首个自主制定的农业类国家标准《热带亚热带桑树栽培管理技术规程》获准发布实施。“国家生猪种业工程技术研究中心”获得批准，成为粤西地区第一个国家级工程技术研究中心。“罗定稻米”和“云安蚕茧”申报通过国家质检总局审查，国家地理标志保护产品达13个。LED照明产品推广应用工作扎实推进，全市新装LED公共照明路灯5779盏，改造公共照明路灯2.55万盏。旅游事业稳步发展。全年接待国内外游客1619.54万人次，增长44.11 %；实现旅游总收入157.95 亿元，增长26.43%。新兴县被国家农业部、旅游局认定为全国休闲农业和乡村旅游示范县。

平安云浮建设深入开展。社会组织管理体系改革进一步推进，新登记社会组织94个。“弘扬优秀传统文化促进社会管理创新”项目获“广东治理创新奖”，自然村乡贤理事会培育和发展基本实现全覆盖。在镇（街）设立政务服务中心，在行政村设立便民服务站，全面提升服务农村水平。全面推进平安云浮建设，实施打防管控建工作。深入开展“平安细胞”工程建设，认真做好矛盾纠纷和不稳定因素排查化解工作，调解率100%、成功率99.2%，受理群众来信来访件（人次）同比下降18.78%，全国全省“两会”期间，群众进京非正常上访量为零，受到省通报表扬。严厉打击“三违三抢”行为，组织了33次大型联合执法行动，拆除违法建设面积2.4万平方米，清理抢种青苗34.7万棵。深入开展打黑除恶、禁毒、打击盗窃摩托车等专项行动，在市中心城区创新交巡警合一的勤务运作模式，加快群防群治步伐，各类违法犯罪警情同比下降15.5%，打掉各类犯罪团伙173个，没有重大刑事案件发生。加快推进社会治安视频监控系统二期建设，以信息化为引领提升公安机关核心战斗力和情报主导警务能力。安全生产形势持续稳定，没有发生重大安全生产和食品安全事故。加强应急管理，没有发生重大群体性事件。社会大局持续稳定，群众安全感进一步提升。

——政府自身建设继续加强。

坚持依法行政，努力打造阳光政府、人民满意政府。认真执行市人大及其常委会各项决议并报告工作，主动向市政协通报情况，广泛听取民主党派、工商联、无党派人士和各人民团体意见，共办理人大代表建议84件、政协委员提案178件，向市人大常委会报告生态建设、教育创强等事项26项，向市政协通报经济社会发展情况、交通建设等事项12次。严格落实中央“八项规定”和省的实施办法，认真查摆和整改“四风”等方面存在的突出问题，坚持立行立改。加强政府一般性支出管理，严格执行“三公”经费的各项规定。建立作风建设全方位监督平台，开展正风肃纪行动。建立改进作风、联系群众长效机制。市政府的会议、文件、简报均大幅减少，考核检查活动压减到原有的10%以内，撤销评比达标表彰活动19项。充分发挥行政效能投诉中心作用，开通了网络行政效能投诉平台。效能监察、廉政建设和反腐败工作不断加强，查处了一批违纪违法案件。政府职能和机关作风进一步转变。

此外，审计、物价、统计、人事人才、经济发展研究、项目代建服务、民族宗教、档案、外事侨务、港澳事务、台务、人民防空、地方志、消防、保密、残联、侨联、文联、社科联、妇儿、青年、工商联、红十字会、贸促会、新闻出版以及驻市外机构等工作也取得新成绩。

各位代表！过去一年政府工作取得的成绩，来之不易。这是在省委、省政府和市委的正确领导下，全市上下开拓进取、奋力拼搏的结果。在此，我代表市人民政府，向全市人民、驻云浮部队官兵和关心支持云浮发展的港澳台同胞、海外侨胞及国际友人表示衷心的感谢并致以崇高的敬意！

同时，我们也清醒地看到，我市经济社会发展还存在一些亟待解决的突出问题。主要是：经济总量不大，财政自给水平比较低；三产业发展不够协调，经济转型压力较大；基础设施不够完善，公共服务体系不够健全；社会保障能力水平不够高；转变政府职能任务繁重。等等。对此，我们将高度重视，采取有效措施切实加以解决。

2014年工作安排

各位代表，2014年是全面贯彻落实党的十八届三中全会精神、全面深化改革的重要一年，是完成“十二五”规划目标任务的关键一年，做好今年的工作意义重大。当前，省委、省政府促进粤东西北地区振兴发展给云浮带来的机遇前所未有，佛山市对口帮扶我市的力度前所未有，全市上下合力谋发展的干劲前所未有，只要我们把握好机遇，进一步加快融入珠三角并开展更高层次的合作，就一定能在新的起点上推动全市经济社会发展实现新跨越。

今年工作的总体要求是：坚持以邓小平理论、“三个代表”重要思想、科学发展观为指导，全面贯彻落实党的十八大、十八届三中全会、中央经济工作会议、省委十一届三次全会和市委五届五次全会精神，以改革促发展，以发展惠民生，突出优化发展环境，加快转型升级，促进城市扩容提质，努力提升经济发展速度，切实提高质量和效益，保持社会和谐稳定。

今年的主要预期目标是：地区生产总值增长15%；人均地区生产总值增长14.4%；地方公共财政预算收入增长22%；规模以上工业增加值增长29%；固定资产投资额增长30%；社会消费品零售总额增长14%；居民消费价格涨幅在3%左右；外贸进出口总额增长8%；城乡居民人均收入增长15.5%；单位GDP能耗下降3%。

为实现上述目标,今年政府工作主要做好以下几个方面，具体为“突出三个重点，打好三大会战，深化三项改革，抓好三大工程，加强三项建设”：

一、突出一产求优、二产求大、三产求快的三个发展重点，努力提升经济发展质量和效益

坚定不移推进产业转型升级，加快构建支撑云浮当前和未来发展的现代产业体系，着力提升经济发展质量和效益，促进一二三产业全面协调发展。

（一）优化发展现代农业。高度重视“三农”工作，扎实推进特色农业建设，提高农业综合效益，提升农业产业化水平。加快建设高标准基本农田，改造传统农业，发展现代农业。深入开展粮食稳定增产行动，建设连片大规模粮食生产示范区，稳定粮食播种面积达180万亩、粮食总产量71万吨以上。推进中草药、品牌蔬菜、花卉苗木、高产油茶等特色种植业规模化、标准化生产。发展农业生态观光等乡村旅游休闲农业。加快培育农业龙头企业、专业大户、家庭农场等新型经营主体，培育市级重点农业龙头企业8家以上，新增农民专业合作社500家以上。加强农产品质量监管，创建国家农产品质量安全监管示范市。推进农田水利工程建设，抓好中小河流治理、病险水库除险加固、中小型灌区续建和节水改造等项目建设。扎实抓好新兴、郁南创建省小型农田水利重点县工作。推进农业科技创新，组织实施“广东农村信息直通车工程”，提升基层科技服务能力。

（二）做大现代工业产业。大力提升传统支柱产业，推动“五大产业”逐步向“千亿产业”目标发展。更加突出发展高新技术产业，推进中小企业孵化器和创新产业示范基地建设，重点帮扶高成长性中小企业和科技型中小企业；加快软件园、科技园建设，不断提升高新技术产业比重。进一步提升装备制造业成套设备制造能力，加快推进汽配产业基地建设。大力培植和发展物联网、现代物流、保健养生、生物医药、新材料、新能源、3D打印技术等产业。加大技术改造力度，提升纺织、食品、日用化妆品等行业，发展包装印刷、工艺美术等都市型工业。加大石材行业整治，优化布局，促进石材行业健康发展。充分发挥国家石材产品质量监督检验中心的作用，提升云浮石材产业的竞争力。继续实施“还珠工程”，加强对中小微企业的服务和管理，促进中小微企业和个体工商户转型升级，做大产业规模。努力推动对外贸易转型升级，积极开拓东盟贸易市场，努力推动主要产品出口种类和数量实现新突破。做大跨境人民币结算规模，提升对外贸易质量。抓住国家加快“珠江—西江经济带”建设的机遇，扩大与相关地区的合作。

（三）提速发展现代服务业。加快推进大型房地产、大型商贸广场等重点项目建设。加强城市新区农贸市场规划建设，支持各县（市、区）建设果蔬、鲜活农产品等专业物流市场。优化城乡商业布局，加强对美食、购物、休闲等特色商业的规划和引导，推进“万村千乡市场”工程建设。加快发展科技、信息、金融、动漫等生产性服务业。以云浮石材博览中心和云浮石材产业城为依托，做大云浮石材网、石汇网等电商平台。抓好智慧城市试点和物联网应用，推进光纤入户和WiFi热点覆盖。推广新业态应用拓展和三网融合战略性新兴产业集聚发展，继续推进省级IPTV平台发展和全市三网融合双向网络建设覆盖。全面提升商贸、餐饮、物业管理等生活性服务业，积极培育文化创意、会展经济等新兴服务业。大力发展新型消费和绿色消费，挖掘农村消费潜力。加大重点旅游项目的招商引资力度，着力打造区域性旅游目的地，加快提升景区品质和等级。积极开展“智慧旅游”试点城市工作，推进与珠三角及两广地区旅游的开放合作。

二、打好交通基础设施建设、园区扩能增效、城市扩容提质三大会战，努力增强加快发展后劲

深入开展“会战攻坚年”活动，突出打好三大会战，完善基础设施，打造发展平台，优化发展环境。今年继续安排108项重点工程（项目），计划总投资1930.84亿元，年度计划投资350.05亿元。

（一）优化立体交通体系。今年交通计划投入75.33亿元。重点抓好南广铁路、江罗高速公路、阳罗高速公路、高村立交，云浮新区东部快线、西部快线、云杨公路、沿江公路等在建项目建设，确保南广铁路、云杨公路一期第二和第三标段、高村立交等一批项目年内建成通车。全力加快汕湛高速云浮段、思劳立交、云浮新区互通立交、省道S369线罗定段路面改造工程、省道S279线郁南县都城至宝珠段改造工程、南广铁路云浮东站交通枢纽、云浮新港物流园、西江星晖造船厂码头、云浮郁南南江口码头扩建等一批项目动工建设。大力推动云浮（罗定）航空产业园建设。抓好三茂铁路云浮高峰至云浮新港铁路专线工程、国道324线云浮市腰古至茶洞段改线工程的前期工作。做好汕湛高速公路新兴至湛江段、怀罗高速公路、罗信高速公路、西江发电厂煤专用码头等项目储备。谋划西江航道整治、广梧高速公路扩建、三茂铁路（含阳春至罗定支线和罗岑铁路）升级改造、广明高速公路西延长线与江罗高速对接、珠三角至云浮轻轨、新城区游艇码头等一批项目。落实建管养并重措施，着力提升国省道和农村公路等级，建设农村公路420公里，提升公路养护和运输水平。推动城市公交优先发展，完成公交发展规划，加快环保、低碳城市公交快速运输通道建设，推进公路、水路、铁路、航空综合运输体系建设，改善交通出行条件。

（二）提升园区承载水平。积极做好佛山对口帮扶我市的对接合作工作，完善组织架构，创新园区投资建设运营机制，力争在园区建设、产业转移、产城融合等方面的深度合作取得新突破。积极推动广佛肇云区域经济一体化，融入珠三角借力发展。加大园区招商引资和项目建设力度，完善园区基础设施和金融、教育、医疗、交通、通讯、消防、商贸、文化娱乐等公共服务配套设施建设；完善园区考核机制，加强对园区规划实施、项目建设、达产达标、质量效益和基础配套进度情况的考核。结合新区产业规划，加快佛山（云浮）产业转移工业园各项规划编制。加快南园主要道路、供电供水、排污设施、美化绿化等规划建设，重点发展汽车配件、机械制造、新型建材、医药食品加工等产业。加快顺德（新兴新成）产业转移工业园的招商引资及扩园申报；进一步完善六都分园的道路、变电站和污水处理厂等基础设施；双东分园要尽快建成“两纵两横”主干道和物流园骨干路网，并争取通过省级产业转移工业园批准认定。规划建设中意（云浮）产业园。力争全市产业园区实现工业产值500 亿元，工业增加值130亿元。力争重点产业园区和重点产业年内完成招商引资800亿元，实际投入资金100亿元。

（三）加快城市扩容提质。加快云浮新区核心区开发建设。抓好南广高铁综合交通枢纽、华南师范大学附属云浮学校、西江新城医院、远大酒店、金融街、特色小镇、农民集中安置小区等项目建设，加快宝能、敏捷、奥园、碧桂园、大润发等城市综合体项目建设。不断完善新区道路交通、卫生教育、娱乐休闲、供水供电、防洪排涝等基础设施建设，进一步完善新区配套设施。加快行政区划调整，争取年内实现云安县撤县设区。大力推进云浮城区的提质，推动各组团与中心城市同城一体化。大力发展公共交通，加快停车场、健身休闲场地、应急避难场所等公共配套设施建设。抓好中心城区和各县（市）城区主干道改造、市政道路贯通、小街小巷整治工程，进一步拉通、拓展城区路网，缓解交通压力。扎实推进市城区蟠龙天湖、英东体育馆广场改造工程。大力开展城市绿道、沿江绿带、社区公园建设，基本达到生态文明城市标准。进一步加大环境整治力度，着力推进生态绿色发展，巩固和提高创建国家卫生城市和省文明城市工作成果。积极开展卫生县城、文明县城、卫生镇、卫生村和文明村镇创建工作，全面提升城乡环境卫生整体水平。进一步扩大罗定、新兴和郁南的城区规模。积极稳妥推进新型城镇化，加强规划，优化布局，有序推进农业转移人口市民化。加强重点镇、中心镇建设，打造一批功能完善、特色鲜明、山水风貌的美丽城镇。推动城镇基础设施和公共服务设施建设向农村延伸，推进城乡一体化发展，促进城乡要素平等交换和公共资源均衡配置。

三、深化经济领域、农村发展、社会管理三项改革，努力激发发展活力

积极落实省关于今年重点领域13项改革的部署，深入开展“改革深化年”活动，着力处理好政府和市场的关系，努力优化政务环境、要素环境、市场环境、信用环境、营商环境和金融环境，营造环境高地。

（一）加快经济领域改革。抓住制约经济社会发展的突出问题，加大市场化改革攻坚力度，更好发挥市场在资源配置中的决定性作用和政府主导作用。切实抓好公共资源交易中心建设，逐步健全资本、产权、技术、知识产权等交易市场，促进要素市场投资主体多元化，建设统一开放、竞争有序的市场体系。推进商事登记制度改革，以优化行政审批流程及标准化建设工作为重点，不断深化行政审批制度改革，放宽市场准入限制，降低准入门槛。理顺政府与市场、社会的关系，理清明晰部门审批监管职责。充分发挥各级网上办事大厅的作用，实现90%行政审批事项、80%公共服务事项网上办理。创新城市建设投融资机制，探索BT、BOT以及“四位一体”的融资模式，吸引更多金融资本参与中心城区扩容提质。创新园区投资建设运营机制，通过市场化运作、股本比例分享收益的形式，吸引更多社会资金参与园区建设。深化“三规合一”，提高科学统筹利用资源的水平。

（二）深化农村改革。全面加快建设农村改革试验区。不断创新现代农业经营体制机制，扶持发展壮大温氏模式和罗定“聚龙”模式，加大探索“公司+现代家庭农场”、“公司+合作社+农户”、“合作社+合作社”三大新型经营模式力度，着力构建集约化、专业化、组织化、社会化相结合的新型农业经营体系。深入推进信用云浮建设，突出抓好重点项目，构建社会信用体系和市场监管体系，全面推进两建工作。建设覆盖全社会的公共信用信息管理系统，建立信用分类监管、经营异常名录和黑名单制度。加快金融改革创新步伐，加快建设农村金融改革创新综合试验区。推动云城区、罗定市农村信用联社改制为农村商业银行，力争增设1-2家村镇银行。加大国内外金融机构引进力度，年内争取落实2家以上金融机构进驻我市。加快培育资本市场，推动温氏集团整体上市及设立企业集团财务公司。探索试行“政银保”合作模式涉农贷款，鼓励发展涉农保险，逐步建立广覆盖的涉农保险保障体系，增强农业抵御自然灾害能力。

（三）创新社会治理。进一步探索构建多元共治体系。整合现有资源，将弘扬优秀传统文化、信用云浮建设、培育发展自然村乡贤理事会和社会组织等统一起来，构建多元主体参与的社会治理体系，建立健全信用政府、信用企业、信用村、信用个人和农村基层社会组织积极协同，乡贤和群众广泛参与，协同共治的社会治理机制。推进构建枢纽型组织体系、社会组织登记管理体制改革、强化社区服务功能、建立镇村便民代办服务机制、探索创新新区社会管理机制等社会体制改革项目建设，确保社会体制改革取得实效。进一步推进城乡基层自治。创新农村（社区）基层社会管理和服务体制，引导发展农村（社区）社会组织，鼓励和支持社会各方参与，实现政府治理和社会自我调节、居民自治良性互动。同时，探索创新住宅小区物业及社会治理方式，创新外来人口管理手段。

四、抓好生态文明、社会保障、民生实事三大工程，全面构建美丽幸福新云浮

按照建设生态文明发展示范区的要求，切实保护好云浮的青山绿水。更好地提升民生福祉,满足广大群众的民生新需求，促进社会和谐发展。

（一）实施生态文明工程。深入实施“森林碳汇、生态景观林带、森林进城围城、乡村绿化美化”四大重点生态工程，重点推进城市绿道、城市公园、江河沿岸和高速公路、南广铁路两旁绿化景观建设。以消灭宜林荒山、改造疏残林、纯松林和布局不合理的桉树林为重点，建设森林碳汇林13.76万亩，推进4.2万亩新老城区林相改造和新区森林公园建设。抓好环境保护，大力发展循环经济，全面推进工程减排、结构减排和管理减排，确保主要污染物持续均衡削减。严守耕地保护红线，促进土地节约集约利用，强化矿产资源管理，提高土地利用率。严格执行投资强度、土地规模控制、产出贡献的刚性标准，鼓励推进高层标准厂房建设。健全自然资源产权制度和用途管制制度，对森林、山岭、荒地、滩涂等生态空间进行统一确权登记。实施重大生态修复工程，重点抓好天然林、生态公益林保护和退耕还林、沿江防护林和水生动物养护工程建设。加强城乡水环境和空气质量监管，抓好城市水源地和重点城镇备用水源工程规划建设，加大乡镇集中式饮用水水源地保护力度。推动各县城、各镇（街）建设一个森林公园。启动幸福村居创建行动，加快城镇污水处理厂建设，完善“户收集、村保洁、镇集中、县（市、区）转运处理”的农村生活垃圾处理模式，全面改善农村人居环境。城镇生活污水集中处理率达到73.8 %（其中市区85%）、城市生活垃圾无害化处理率达到100%。落实干部挂钩制度，继续推进生态镇村建设。

（二）实施社会保障工程。推进就业惠民、创业富民、投资兴民、社保安民、扶贫助民。健全促进就业创业机制，城镇新增就业3万人、农村劳动力转移就业3万人，扶持创业1200人，带动就业5000人。逐步完善各项社保制度。探索城乡居民社会养老保险“多缴多得”长效机制，基础养老金提高到80元。制定特困参保人员养老保险补助办法，加强大病救助。完善新型社会救助体系、适度普惠型社会福利体系、社会养老服务体系和优抚安置工作体系。保障困难群众尤其是特困群体基本生活，完善能托底的社会保障制度。强化土地被征收农民社会保障工作，推动异地务工人员工伤、失业保险全覆盖。扎实推进和谐劳动关系示范区建设，切实化解劳资纠纷。

（三）实施民生实事工程。今年继续重点办好“十件民生实事”，让人们享有更高水平的民生保障、医疗卫生、教育文化服务。主要体现在：一是全面推进教育强市建设。年内创建15个省教育强镇，抓好云城区和新兴县创建教育强县（区）和义务教育基本均衡县工作。大力推进义务教育均衡优质标准化发展。积极推进教育信息化建设。实施西江新城教育发展计划，扎实推进华南师范大学附属云浮学校建设。大力推进校园安全工程，全市95%以上学校达到“平安校园”要求。开展现代远程成人教育，推进开放大学建设。提升邓发纪念中学办学质量。积极扩大罗定职业技术学院办学规模，推动规划建设幼儿师范分院。推进市中专学校创建省示范性学校工作。推进规范化幼儿园建设，促进学前教育优质发展。二是推进公共文化体系建设。进一步深化文化体制机制改革，完善市、县、镇、村四级公共文化设施网络，全面推进基层文化设施全覆盖工程。加强文物保护、非物质文化遗产保护和传承，推进各县（市、区）文化生态保护实验区建设。推进创建平安文化市场，深入开展扫黄打非工作。加快推进市体育公园和儿童活动公园建设，新建乡镇农民体育健身广场12个。三是深化卫生体制改革。继续深化医药卫生体制改革，巩固完善基本药物制度，健全基层医疗卫生机构运行机制。继续推进平价医疗服务，每个县（市、区）各建设一家平价医院，100%的公立基层医疗卫生机构使用平价药包。加快推进县级公立医院改革，确保完成省下达的“80%的县级公立医院完成改革”的工作任务。启动实施区域卫生信息化建设。大力扶持民营医疗机构发展。巩固发展中医药强市成果，推动“三甲”中医院和中医“治未病”工程建设。完成农村改厕1万户。深化人口计生综合治理，落实免费孕前优生健康检查等惠民政策，稳定低生育水平。四是全力推进科技创新。推进市科技（科学）中心建设，认真做好LED照明产品推广使用，抓好科技服务业发展、专业镇转型升级和农业科技园区建设等工作。五是切实加强社会服务平台建设。完善社区、社会组织和社工“三社联动”工作机制，健全社区网格化管理体系，建立覆盖社区居民和流动人口的综合性服务平台。扎实推进平安云浮建设，以打防管控建为抓手，继续推进“平安细胞”工程，构建社会治安网格化防控体系，严厉打击各类违法犯罪活动，不断提升群众安全感和满意度。全面落实安全生产责任，有效防范和坚决遏制重特大安全事故发生。加强应急体系建设，推进市、县突发事件预警信息发布中心建设，进一步提升防灾减灾能力。六是加强食品药品安全监管。积极实施食品安全行动计划，保障人民群众饮食用药安全。实施标准化战略，强化打假工作责任制的落实，加强质量强市建设。同时，深入开展第三次全国经济普查和全市城乡一体化住户调查。大力弘扬新时期“诚信、包容、实干”的云浮精神，组织开展社会公德、职业道德、家庭美德和个人品德教育。

继续做好拥军优属、国防教育、人民武装、人事编制、民族宗教、外事侨务、港澳台事务、经济发展研究、机关事务、妇女儿童、项目代建服务、人防、消防、国资、物价、统计、科普、气象、史志、档案、老龄人、保密、残联、侨联、文联、社科联、工商联、红十字会、贸促会、新闻出版以及驻市外机构等工作。

五、加强民主法治、工作作风、勤政廉政三项建设，全面建设人民满意政府

深入开展“作风整顿年”活动，加快职能转变，加强政府自身建设，努力建设人民满意和更加信赖的政府。

（一）全面推进民主法治。进一步规范行政执法行为，加大行政执法监督力度，提高依法行政水平。认真落实市人大及其常委会的决议和决定，加强与市政协的工作沟通和协商。健全行政监督机制，认真听取各民主党派、工商联、无党派人士和人民团体的意见，自觉接受人民群众和新闻舆论监督。争取全省电子政府建设试点。全面推进电子纪检监察综合平台建设。继续落实《云浮市贯彻落实〈法治广东建设五年规划（2011-2015年）〉的实施意见》，做好市政府规范性文件制定工作，完善规范性文件、重大决策合法性审查机制。建立科学的法治建设指标体系和考核标准。健全规范性文件备案审查制度。建立公共法律服务体系，加大政府购买法律服务工作，进一步落实全市行政机关规范行政处罚自由裁量权工作和行政执法责任制。加大行政复议力度，健全行政复议审理机制，纠正违法或不当行政行为，全面推进法治政府建设。

（二）切实转变工作作风。深入贯彻落实中央“八项规定”，扎实开展群众路线教育实践活动，全力解决“四风”突出问题，建立联系群众、改进作风常态化制度。健全领导干部带头改进作风、深入基层调查研究的机制，完善直接联系和服务群众制度。建立市县镇村四级政务服务体系。精简会议和文件，改进会风文风，力戒形式主义、文牍主义。严格控制各类庆典、多重检查、评比及达标创建活动。严格落实公务接待、公车管理和公务考察的监督，杜绝铺张浪费。开展明查暗访，落实工作督查查办、限时办结等制度。深化正风肃纪行动，加强各级政府执行力建设，着力治庸治懒治散治慢治奢，坚决整治“吃拿卡要”、权力寻租等行为，推动机关作风进一步好转。

（三）深入抓好勤政廉政。始终高度重视党风廉政建设，深入推进廉政风险防控管理，全面落实党风廉政建设责任制，加大从源头上预防和治理腐败力度，构建全市动手抓党风廉政建设和反腐败工作的良好局面。建立健全市、县、镇三级公共资源交易中心。严格落实行政问责制度，加强重点项目、土地和房屋征收廉政监督与效能监察。建立地方各级政府债务风险防控和预警机制，严格政府举债程序，加强地方债务审计。加强经济责任审计，强化审计监督。继续加大查办案件工作力度，坚持以零容忍态度惩治腐败，着力解决发生在群众身边的腐败问题，切实做到干部清正、政府清廉、政治清明，形成为民务实、清廉高效的良好政风。

各位代表，“空谈误国，实干兴邦”。让我们在中共云浮市委的坚强领导下，凝心聚力、开拓创新、砥砺奋进，为全面建成小康社会，为全市人民过上更加幸福的生活,作出新的更大的贡献！
